# A Smart Textile Biofeedback Training System for Upper Limb Rehabilitation After Stroke: Co-Design Development and Evaluation Study

**DOI:** 10.2196/77999

**Published:** 2026-04-13

**Authors:** Maria Munoz-Novoa, Li Guo, Anna Björkquist, Morten B Kristoffersen, Peiman Khorramshahi, Leif Sandsjö, Margit Alt Murphy

**Affiliations:** 1 Department of Clinical Neuroscience, Institute of Neuroscience and Physiology, University of Gothenburg, Gothenburg, Sweden; 2 Department of Work Life and Social Welfare, Faculty of Caring Science, Work Life and Social Welfare, University of Borås, Allégatan 1, Borås, SE-501 90, Sweden, 46 33 435 4630; 3 Department of Textile Technology, Swedish School of Textiles, University of Borås, Borås, Sweden; 4 Chalmers Industriteknik, Gothenburg, Sweden; 5 Department of Engineering Technology, Technical University of Denmark, Ballerup, Denmark; 6 Daralabs AB, Gothenburg, Sweden; 7 Design & Human Factors, Department of Industrial and Materials Science, Chalmers University of Technology, Gothenburg, Sweden; 8 Department of Health and Rehabilitation, Institute of Neuroscience and Physiology, University of Gothenburg, Gothenburg, Sweden

**Keywords:** stroke, surface electromyography, sEMG, surface electrodes, textile electrodes, smart textiles, upper limb function, self-administered, rehabilitation, co-design

## Abstract

**Background:**

An increasing number of rehabilitation technologies are being developed to support upper limb rehabilitation after stroke, with smart textile solutions for surface electromyography (sEMG) emerging as a promising approach. Early end-user involvement is crucial for developing user-friendly and clinically valid rehabilitation tools.

**Objective:**

This study aims to refine and evaluate the prototype design and usability of a smart textile biofeedback system for self-administered upper limb training after stroke.

**Methods:**

The training system includes a knitted smart textile sleeve with integrated electrodes over the forearm muscles, an sEMG unit, and tablet-based biofeedback software. An iterative co-design process was followed, including initial testing, demonstration sessions with end users (9 clinicians and 10 individuals with stroke), and a final evaluation of the co-design process. Participants’ experiences were gathered through semistructured interviews, analyzed using content analysis, and the User Experience Questionnaire. The co-design team included experts in stroke rehabilitation, textile engineering, biomedical engineering, software development, and human factors, as well as a research partner with lived experience after stroke.

**Results:**

The perspectives of the end users and the expert team were collectively integrated into prototype refinements of the sleeve and training software to meet the needs of the intended target group. The experiences of end users formed 2 main categories: “This could be an exciting new training tool for stroke rehabilitation” and “The tool works well, but some changes could enhance independent training.” End users found the smart textile sleeve and biofeedback system easy to use and saw potential for integrating it into their training routines. Both end-user groups rated the system as attractive, stimulating, and novel.

**Conclusions:**

The results of this study establish a necessary ground toward the development of a smart textile sEMG biofeedback system for self-administered upper limb training after stroke. Findings from the co-design process support the continued development and evaluation of the system as a self-administered upper limb training tool for individuals living with stroke.

## Introduction

Regaining upper limb function is a primary goal for individuals after a stroke [1]. In the acute phase, about 50% to 70% of individuals experience impaired arm and hand function, and in the long term, only a smaller percentage fully recover their previous level of function [2-5]. Upper limb impairment often leads to difficulties in performing daily activities and limitations in participation [6,7]. Most improvements occur early in recovery, with slower progress continuing into the chronic phase [5,8]. To follow this recovery pattern, rehabilitation programs are mostly concentrated in the acute and subacute phases, while in the chronic phase, the responsibility for continued training often falls on the individuals themselves [9,10]. To support long-term rehabilitation [1], various rehabilitation technologies have been developed to facilitate independent home-based training, ultimately aiming to improve functioning [11,12].

Surface electromyography (sEMG) biofeedback is a rehabilitation technology that provides real-time visual feedback on muscle activity, thereby supporting motor learning mechanisms for more effective movement execution [13,14]. Previous research in stroke populations indicates that sEMG biofeedback interventions can increase upper limb muscle strength, active range of motion, and awareness of the paretic arm [15-18]. sEMG biofeedback systems commonly use disposable pre-gelled Ag/AgCl electrodes [19]. As an alternative to these traditional electrodes, smart textile solutions with integrated textile-based electrodes offer improved comfort, easier application, and a reduced risk of skin irritation [20-24]. Textile solutions enable prolonged, repeated use, which makes them suitable for self-administered rehabilitation in both clinical and home settings. However, smart textile solutions for sEMG applications in upper limb stroke rehabilitation are in the early stages of development, with existing studies limited to controlled research environments and/or small sample sizes [25-27].

A challenge in developing new rehabilitation technologies is their implementation in real-world settings [12,28,29]. A failure to meet the needs of end users (patients and clinicians) and underestimating the complexity and costs of implementation in clinical practice are common reasons for limited clinical uptake [30-32]. Adopting collaborative co-design methodologies that involve end users in the development process can help identify and address specific user needs early on. This ensures better alignment among the technology, its intended use, and context and has been shown to improve the applicability and acceptance of rehabilitation technologies [30,33,34].

This study aims to refine and evaluate the prototype design and usability of a smart textile biofeedback system for self-administered upper limb training after a stroke, using a co-design approach.

## Methods

### Ethical Considerations

The study was approved by the Ethics Review Authority (reference 2019-00450/1074-18 and amendment 2024-01703-02) and follows the COREQ (Consolidated Criteria for Reporting Qualitative Research) checklist (Checklist 1) [35]. Before entering the study, all participants completed an approved informed consent form, as authorized by the Swedish Ethics Review Authority, including information about the possibility to withdraw from the study at any time without providing a reason. All collected data were anonymized and safely stored accessible only to the research team. No compensation was given for participation.

### Co-Design Process

**Figure 1.**
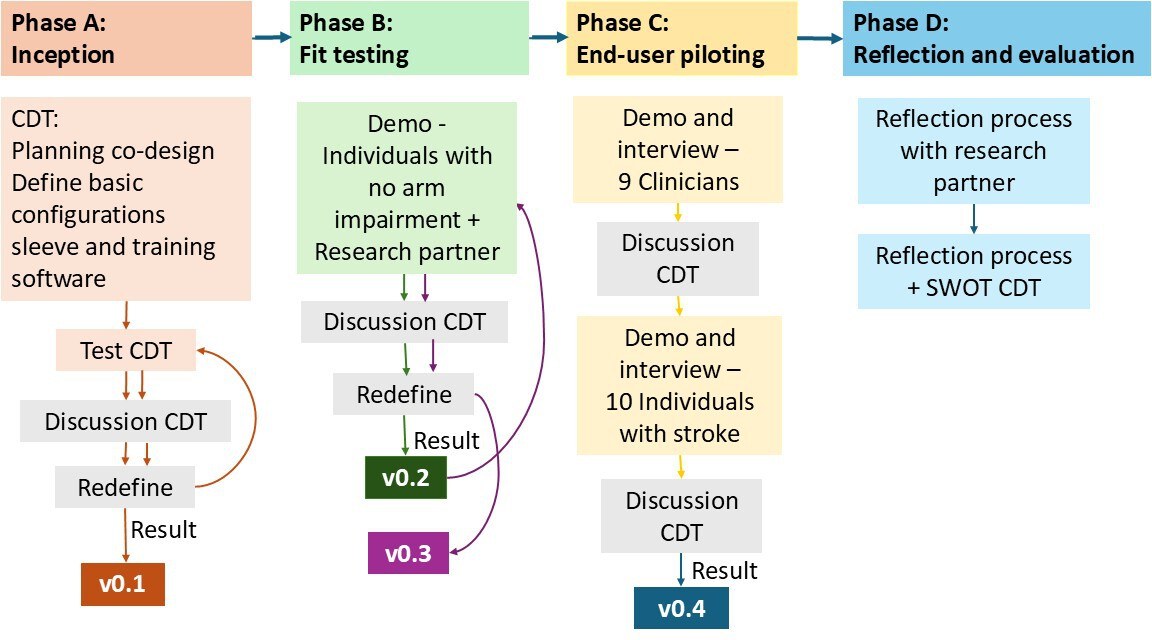
The 4 phases of the co-design process, guided by the participatory action research model and the Medical Research Council framework, showing the iterations leading to gradually evolving prototype versions (v0.1-v0.4). CDT: co-design team; SWOT: strengths, weaknesses, opportunities, and threats.

The participatory action research model [36] and the Medical Research Council framework for complex interventions [37] were used to guide the iterative and reflective co-design process, which included 4 phases (Figure 1). Each phase involved iterative cycles of problem definition, planning, acting, observing, reflecting, and redefining the problem [36]. At the end of the codesign process, a strengths, weaknesses, opportunities, and threats analysis [38] was conducted to evaluate the co-design process. The aims, procedures, and actions of each co-design phase are detailed in Table 1.

The co-design team included 7 experts from various fields, including stroke rehabilitation, biomedical engineering, textile engineering, signal processing, software development, and human factors. Most team members had over 15 years of experience in their respective areas, including 1 industry representative with a background in commercial product development. The team also included 2 junior researchers, each with approximately 5 years of experience.

A research partner with a university degree and over 5 years of lived experience with stroke was also included in the co-design team to provide end-user insights during the co-design process. Following the iterative approach of the co-design process, regular physical and digital meetings were held within the co-design team between December 2023 and February 2025.

**Table 1.** Co-design process and refinements of the training system.

Phase	Aim	Procedures	Actions
A: Inception	To set priorities for the co-design process and discuss basic configurations for the training system.	The CDT^a^ met several times to set priorities and define the initial configuration of the training system. The CDT also tested the first version of the sleeve and software.	Version 0.1: a conically shaped, circularly knitted sleeve with 2 electrode pairs (wrist flexors and extensors) and a reference electrode at the elbow was developed. The electrodes were connected via snap-button connectors to a wireless sEMG^b^ acquisition unit (Shimmer3 EMG, Shimmer Sensing) with an external band. To enhance signal quality, water was applied by 3 finger strokes to each electrode prior to use. Biofeedback was delivered through a tablet using a custom-made application (iOS and Android compatible). Real-time sEMG signals from 2 channels (wrist flexors and extensors) were used during a calibration period to set training thresholds. For training, a vertical bar (presented next to the sEMG signals) turned green when the user reached a target threshold.
B: Fit testing	To test the training system with individuals without upper limb impairments and refine its design for improved fit and usability.	Individual demonstration sessions with the research partner and with 11 participants without arm impairments. Refinements and production of the sleeve.	Version 0.2: sEMG signals were obtained from all participants, although the comfort of the sleeve varied across participants. Based on their feedback, medium and large sizes were produced, and a reference line following the middle finger was added to facilitate electrode alignment during donning. Version 0.3: a second feedback round led to further refinements, including a knob for easier elbow alignment, a Velcro strap to attach the sEMG unit to the sleeve (replacing the external band), and the addition of a small sleeve size. Software updates included adding a separate window for visualizing the bar activity, tracking repetitions exceeding the threshold, and implementing calibration with a manual stop.
C: End-user piloting	To assess usability and investigate perceptions and experiences of the training system from clinicians and individuals with stroke.	Individual demonstration sessions with 9 stroke rehabilitation clinicians and 10 individuals with stroke. Semistructured interviews and the UEQ^c^ with end users at the end of the demonstration sessions.	Version 0.4: on the basis of the collected feedback by end users, an additional Velcro strap was added to the sleeve to secure cables and simplify donning and doffing. The medium-sized sleeve was also downsized for a better fit. Software improvements included: fewer and larger buttons, naming of channels, adding a calibration countdown, larger size of the bars with repetition counts, higher color contrast, and a performance report.
D: Reflection and evaluation	To reflect on and to evaluate the co-design process.	SWOT^d^ analysis and reflection workshop with CDT. Individual reflection session with research partner.	Reflections from the CDT highlighted strengths such as strong collaboration, research partner involvement, and interdisciplinary expertise. Weaknesses included lack of an external facilitator and limited access to in-house software developers. Opportunities and threats related to future implementation were discussed. The research partner provided additional insights on her experience and valued her active role throughout the co-design process.

a CDT: co-design team.

b sEMG: surface electromyography.

c UEQ: User Experience Questionnaire.

d SWOT: strengths, weaknesses, opportunities, and threats.

### Participants and Data Collection

In phase A, the co-design team defined the basic configuration of the training system (version 0.1). A textile sleeve with 2 pairs of integrated textile electrodes, placed over the wrist extensors and flexors, and a reference electrode placed on the elbow was produced. The sleeve was connected to a 2-channel wireless sEMG system (Shimmer3 EMG, Shimmer Sensing). The biofeedback software provided visual feedback of the sEMG signals displayed on a tablet from the wrist extensor muscles (eg, extensor carpi radialis brevis) and flexor muscles (eg, flexor carpi ulnaris). The sEMG signals from specific muscle activation were used to calibrate muscle activity thresholds, which were then visualized during training through a vertical bar display. This provided real-time feedback and allowed for adjustments to individualized muscle activity threshold settings.

In phase B, an initial test demonstration session was conducted with the research partner, followed by individual sessions with 11 participants without arm impairments. Convenience sampling was used, and age, gender, and anthropometric data were collected from this sample (Multimedia Appendix 1).

In phase C, individual demonstration sessions were conducted with 2 end-user groups: 9 clinicians (6 physiotherapists, 2 occupational therapists, and 1 rehabilitation doctor) and 10 individuals with stroke (Tables S1 and S2 in Multimedia Appendix 2). Participants were recruited through purposive sampling. Clinicians were recruited from stroke rehabilitation units in hospitals and primary care centers, while individuals with stroke were recruited through advertisements at rehabilitation centers in the Gothenburg urban area. Inclusion criteria for individuals with stroke were as follows: aged over 18 years, upper limb impairment after stroke, and detectable sEMG signal from the paretic arm. Exclusion criteria for all participants included open wounds, other conditions affecting arm function, uncorrected visual impairment, and inability to understand or follow instructions in Swedish or English. Demographic data, prior sEMG experience, and familiarity with using technologies (eg, smartphones, tablets, and computers) were collected from all end users. For individuals with stroke, additional information was gathered on stroke type and time since stroke, while for clinicians, data on years of experience and work setting were collected.

All demonstration sessions were conducted at Gothenburg University and led by a physiotherapist experienced with the training system (MM-N). In these sessions, the participants were first instructed on how to use the sleeve and software and then asked to try it independently, with additional support provided as needed. At the end of the sessions, the participants were interviewed for about 30 minutes and asked to fill in the User Experience Questionnaire (UEQ) [39,40]. Additionally, in the demonstration sessions with individuals with stroke, upper limb function and spasticity were assessed using the Fugl-Meyer Assessment of Upper Extremity [41] and the Modified Ashworth Scale [42], respectively. From the demonstration sessions with end users, 1 clinician tested only the sleeve due to technical issues with the software and 1 individual with stroke provided limited feedback due to aphasia.

### Evaluations and Data Analysis

#### Semistructured Interviews

The semistructured interviews included questions about the end users’ perceptions and experiences using the sleeve and training software during the demonstration sessions, as well as their opinions on the training tool’s potential for independent use in clinics or home settings. For clinicians, questions were adapted to explore how they would foresee using the training system in stroke rehabilitation (Multimedia Appendix 1). All interviews were led by the same physiotherapist who conducted the demonstration sessions (MM-N, with more than 5 years of research experience in stroke rehabilitation), unless a participant was not fluent in English, in which case the interview was conducted in Swedish by another physiotherapist (MAM, with more than 20 years of experience in stroke rehabilitation). All interviews were audio-recorded, transcribed verbatim by 2 researchers from the co-design team (MM-N and AB), and stored securely, with access restricted to the authors. The field notes and spontaneous feedback collected from participants during the demonstration sessions were also used to complement the interview data.

Content analysis using the approach described by Graneheim and Lundman [43] was used to analyze the interviews [44]. The analysis involved extracting meaning units, condensing them, coding, and sorting codes into categories and subcategories (Multimedia Appendix 1). Since the interviews were conducted following a single demonstration session, the analysis focused on manifest content, emphasizing explicit and observable elements [43,44]. Interviews conducted in Swedish were translated into English and checked for accuracy by a native Swedish speaker (AB). The analyses were conducted in English. Initial coding was carried out by a member of the co-design team (MM-N), while a second researcher (AB), a research engineer with over 5 years of experience in textile technology who was not involved in data collection but was familiar with the data, participated in the review and refinement of the codes. Interviews with clinicians and individuals with stroke were analyzed together, but coding was grouped separately for each participant type using different colors to facilitate category development while distinguishing differences between the groups. Two researchers (AB and MM-N) independently developed initial categories, followed by an iterative process of discussions with a third researcher (MAM) to refine and resolve conflicts until final categories were agreed upon. The research partner was also included in the final stages of the analysis, providing additional input on the selected categories and subcategories.

#### User Experience Questionnaire

The UEQ captures a broad range of user experience aspects, helping to identify both strengths and areas for improvement [39,40]. It consists of 26 items rated on a 7-point scale, with each item presented as a pair of opposite terms. The questionnaire evaluates 6 dimensions: attractiveness (overall impression), perspicuity (ease of use), efficiency (task completion without unnecessary effort), dependability (feeling in control), stimulation (excitement and motivation), and novelty (innovation and creativity) [39,40]. The UEQ benchmark [45] was used to interpret the results on 5 levels: excellent, good, above average, below average, and bad.

## Results

**Figure 2.**
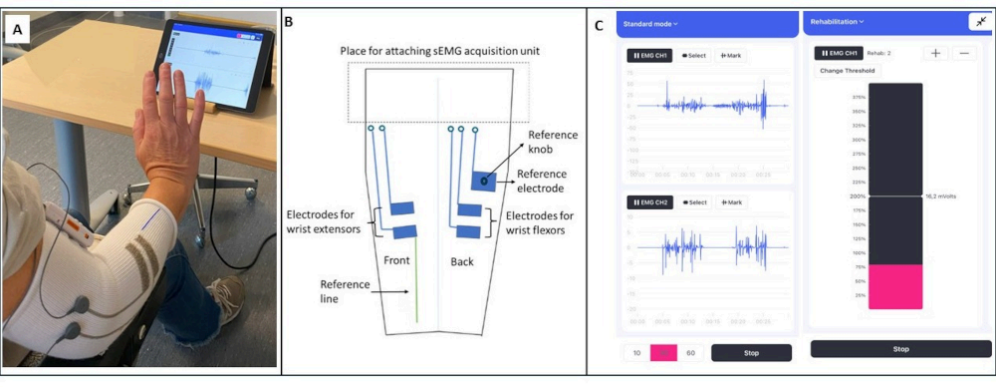
The smart textile sleeve biofeedback training system version 0.3. (A) Demonstration session setup, (B) smart textile sleeve configuration, and (C) biofeedback software (left panel: real-time sEMG signals from extensor [CH1] and flexor [CH2] channels; right panel: muscle activity bar visualization with adjustable threshold settings for training). CH1: channel 1; CH2: channel 2; sEMG: surface electromyography.

The specific refinements of the training system during the iterative phases (A-D) of the study are detailed in Table 1. The setup of the training system, the smart textile sleeve configuration, and the biofeedback software used in the demonstration sessions (version 0.3) are shown in Figure 2.

### End Users’ Experiences and Perceptions

From the interviews with clinicians and individuals with stroke, 2 main categories and 5 subcategories emerged, as illustrated in Textbox 1. Quotes are presented below each subcategory, where “S” indicates individuals with stroke and “C” refers to clinicians.

Textbox 1. **Categories and subcategories emerging from content analysis.**

**Categories and subcategories**

- This could be a new exciting training tool for stroke rehabilitation.
  - Rewarding and motivating to see muscle activity.
  - Use will be determined by individual needs.
  - Instructions and guidance will help independent use.
- The training tool works fine, but some changes could enhance independent training.
  - It can be difficult at first, but it is possible to find a way to make it work.
  - More is needed for independent long-term use.

#### This Could Be a New Exciting Training Tool for Stroke Rehabilitation

Participants with stroke and clinicians found the training system to be novel, exciting, and potentially useful for upper limb rehabilitation after stroke. Both groups expressed interest in incorporating the training system into their routines.

##### Rewarding and Motivating to See Muscle Activity

The real-time visual feedback on muscle activity was described as intuitive, useful [2], and rewarding by most participants. Individuals with stroke liked seeing their muscle activity, especially during training with the bar visualization, as many had thought they had little or no muscle activity due to their impairment. Some participants reported feeling more “connected” to their affected arm when seeing the activity visualized on the screen [3,4]. They described the feedback as not only giving them a sense of control over their arm but also possibly making the training more engaging, interesting, and motivating. Clinicians also highlighted the benefits of visual feedback, particularly for patients with limited muscle activity, as it could help them recognize their remaining muscle function and visualize their progress over time. Participants with stroke and clinicians also noted that visual feedback could enhance motivation and engagement and increase awareness of the affected arm, ultimately supporting and empowering independent training.

***S5:*** *That’s, for me, [seeing the muscle activity] is something good to see, because then I see that something actually happens…I mean, it’s boring to just sit like that, but if you see something happening, then I know my muscles are generating that kind of activity.*

***C3:*** *I like the visual feedback you get, even though we just do like a slight movement, you see the graph is changing. And if I put it on stroke patients, I think it’s really good because sometimes when you tell a stroke patient, “Oh, can you do this movement?” They are quite sad, because they can’t do the movement, but [with this training] you can see, “Oh, they are initiating the movement.”*

##### Use Will Be Determined by Individual Needs

Most participants considered the training system more suitable for the chronic or subacute phases after a stroke. In the acute and subacute phases, individuals with stroke felt it could be overwhelming due to the many other challenges they faced at that stage, a concern shared by the clinicians. Some clinicians also noted that cost, time, and technical constraints could limit its use in clinical settings, while others described the tool as a complement to other training in the subacute phase. Both groups saw it as feasible and beneficial for home training in the chronic phase, especially considering that the available training opportunities are fewer compared to the subacute phase. However, they noted that some level of upper limb function in the paretic arm would be necessary, as high spasticity or limited elbow and finger control could make it difficult to don and doff the sleeve independently. Clinicians also emphasized that a certain level of cognition is required to handle and manage the training without therapist or carer support. Both groups mentioned that limited prior experience with technology could be a potential barrier to using the software.

***S6:** I think it’s a very good idea [the training tool]. Eh, it’s a problem for people like me [individuals with stroke], in the winter, to get out to training places when it’s snowy, so it’s very nice to make it possible to train home as well.*

***C7:** I think if you have one arm [functional arm], you could do it. You still need good cognitive abilities. Maybe not being too old. Some patients who are old are afraid of technological things because they feel uncertain. Even though they can manage… they don’t think they can. It is very different from person to person.*

##### Instructions and Guidance Will Help Independent Use

Individuals with stroke, as well as clinicians, viewed independent home training positively, with individuals with stroke especially finding it exciting and convenient. However, both groups emphasized that clear instructions and support are essential for successful independent use. While they appreciated the simple and clear guidance provided during the demonstration session, they felt additional help with software navigation and sleeve placement would be required. Individuals with stroke generally believed they could train independently or with partial caregiver assistance at home after initial guidance. However, they desired more structured support, including written training protocols, visual and written instructions, in-software reminders, and clinician follow-ups (in person or online) to ensure success. Clinicians also stressed the need for clear, repeated instructions in multiple formats to facilitate adherence. Additionally, clinicians highlighted the importance of hands-on training for patients and caregivers, along with in-person or online follow-ups to monitor tool usage and progress.

***S2:** It was difficult [to place the sleeve independently]. I think it can be even more difficult if you, kind of, get it [the training system] home and don't get to try it on first.*

***C1:** ...They [individuals with stroke] need guidance, as it is right now [the tested version], I think...but otherwise, I think it’s quite easy to use with guidance.*

### The Training Tool Works Fine, but Some Changes Could Enhance Independent Training

Overall, individuals with stroke and clinicians described the sleeve as functional and easy to use, noting that further improvements to the software were needed to promote independent training.

#### It Can Be Difficult at First, but It Is Possible to Find a Way to Make It Work

Most participants found the sleeve easy to use and comfortable, and they appreciated the provided instructions as well as the reference line and elbow knob for guidance. Individuals with stroke and more severe arm impairment experienced some difficulties with getting the hand inside the sleeve, rotating the sleeve into the correct position, and/or pulling the sleeve up on the arm. However, many found strategies by themselves during the demonstration session and believed that, with practice, it would not be a major problem. Another challenge was locating the reference electrode at the elbow and applying water to the reference and flexor electrodes due to a limited range of arm movement and/or sensory impairment. In the end, all participants with stroke were able to place the sleeve correctly, although some needed more time and minor assistance.

Perceptions of the software varied. Younger participants in both groups found the software easier to use, while others described it as a bit complicated and not intuitive. The buttons on the screen were too small and difficult to see for many. In general, most individuals with stroke and clinicians liked that a tablet was used for feedback, as it is larger than a phone but also portable compared to a computer screen. They emphasized that initial guidance and clear instructions on the software would be essential to ensure successful use.

***S7:** Yes, it was difficult to begin with [to put on the sleeve], but I think it’s something you get used to.*

***C6:** To me, it was easy and fine. I don’t know if a patient with a paresis would have difficulties having it drawn up on the middle of the arm …and also, placing it correctly on the elbow might be difficult.*

#### More Is Needed for Independent Long-Term Use

Independent home use was a possibility that many described as feasible, but they suggested improvements for better usability and long-term adherence. To simplify sleeve placement, participants recommended using a mirror or tablet webcam to better view the electrodes on the dorsal side of the arm and the reference electrode. Additional reference points, shorter electrode cables, and a pull strap for easier placement were also suggested. Regarding the software, both participants with stroke and clinicians desired a more user-friendly, engaging, and intuitive interface, with suggestions including games, videos, and motivational elements to make training more engaging. Clinicians emphasized adding functional, goal-oriented tasks and applying resistance to enhance engagement and facilitate training. Fewer and larger clickable buttons, simpler calibration procedures, and adding more colors and contrast were other proposals for software improvements. Clinicians also suggested incorporating audio feedback to guide training and augment visual feedback. Additionally, both groups highlighted the need for customizable training parameters to facilitate sessions, such as calibration time and the number of repetitions, as well as the ability to track progress, which is particularly important for independent training. When considering home training, participants thought that a short daily training session of about 15 minutes would be feasible—short enough to avoid mental fatigue. A clinician also suggested using a bag to keep the sleeve clean between training sessions and noted that multiple sleeves might be needed if one requires cleaning or washing.

***S10:** On the tablet, I think it’s too many knobs [buttons] to press on…It should be easier. One or two presses.*

***C2:** I’m thinking this [the training tool] was easy to use. But maybe adding some extra, like instructions... instructions like how fast you will do the exercise, how many repetitions, how long will I keep it. Maybe adding…like an audio, like a voice, [for example] if you have bad vision.*

### User Experience Questionnaire

**Figure 3.**
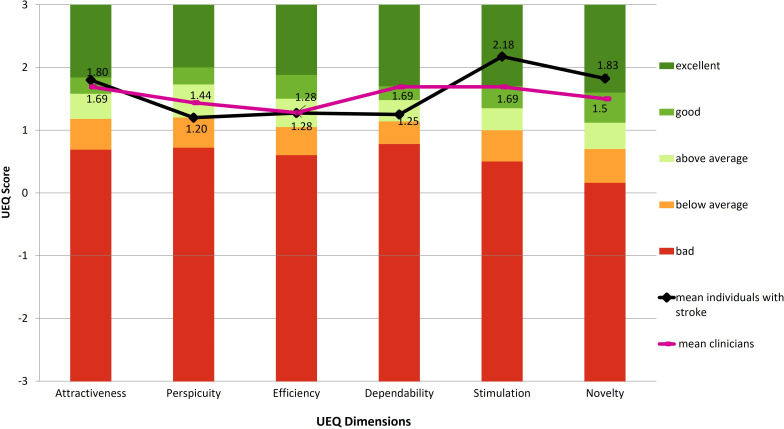
The results from the User Experience Questionnaire (UEQ) in relation to the standard UEQ benchmark levels. Scores range from −3 (very negative evaluation) to +3 (very positive evaluation), with 0 representing a neutral response. Line plots indicate the mean responses from individuals with stroke (black line; n=10) and clinicians (pink line; n=8).

The UEQ was completed by all individuals with stroke and all but 1 clinician, who did not have the opportunity to try the software. Both groups rated *attractiveness* as “good” and *efficiency* as “above average.” Clinicians rated *perspicuity* as “above average,” while participants with stroke rated it lower, between “above average” and “below average.” *Dependability* was rated “good” by clinicians and “above average” by individuals with stroke. Participants with stroke rated *stimulation* and *novelty* as “excellent,” while clinicians rated them as “good” (Figure 3).

### Reflection and Evaluation of the Co-Design Process

The strengths of the co-design process identified by the co-design team were strong collaboration, effective communication, and research partner involvement, ensuring an end-user perspective. Flexibility, team commitment, and optimal facilities were also highlighted. Not having access to an external facilitator and software developers within the co-design team was identified as a weakness, with the latter contributing to delays in communication and iterative development. Opportunities included creating visual simulations before user involvement, direct contact with clinicians and patients, and a positive view of the training tool’s potential value. Threats such as overdevelopment, uncertainty about real-world applicability, and challenges with commercialization and regulatory approval were also identified.

At the end of the co-design process, a reflection session with the research partner revealed that she enjoyed the experience and was pleasantly surprised by how much she liked it, as it was her first time in this role. She appreciated being involved not only in testing the training system but also in the reflective process. Overall, she found the experience fun and felt good about contributing to the research.

## Discussion

### Principal Findings

Supported by collaboration between experts from different fields and end users, the iterative co-design process provided valuable insights into the technical, clinical, and user-centered aspects of the training system, highlighting its potential usability, acceptability, and areas for improvement based on end users’ needs. The participants with stroke were able to independently, or with minor assistance, don and doff the sleeve and operate the training software on the tablet. Both clinicians and individuals with stroke recognized the training tool’s potential for self-administered training. This study provides a starting point for the ongoing development and improvement of the training system.

The structured co-design process in this study, grounded in the participatory action research model [36] and the Medical Research Council framework [37], provided a strong foundation for the development of the training system and the shared understanding necessary for its continued refinement. This approach supported a systematic and iterative development of the training system and may serve as a useful model for future rehabilitation technology development [46,47]. The interdisciplinary nature of the co-design team enriched the process through diverse perspectives. However, differences in disciplinary knowledge and workflows, as reported in previous studies [48-50], may have prolonged the decision-making process. The co-design team suggested that adding a neutral facilitator to the team could have helped to advance the process further [33,51]. Integrating end-user feedback on design improvements with development constraints and limited available resources is a complex but well-recognized challenge in rehabilitation technology development [36,52]. This challenge was addressed through regular co-design meetings and systematic documentation, which enabled balanced prioritization of modifications based on both end-user feedback and practical constraints. Even though not all suggestions from the end users were integrated, they offered valuable insights for future improvements to the training system.

The overall positive perceptions of the smart textile biofeedback training system among individuals with stroke and clinicians underscore its potential as a possible solution for self-administered rehabilitation at home and in clinical contexts. Initial difficulties with donning and doffing the sleeve, particularly among participants with more severe upper limb impairments, highlighted the importance of user-centered design and individualized support during the development of the training system [30,33,34]. All participants were ultimately able to use the system independently or with minimal assistance, suggesting its accessibility across varying levels of motor function. The integration of textile electrodes simplified the donning process, possibly reducing barriers associated with conventional electrode placement and supporting independent use [20-24], especially for individuals with limited fine motor control. Although the system was evaluated in single sessions, the positive perceptions and willingness to incorporate it into daily training routines indicate promising initial usability and acceptability. Further longitudinal studies are needed to assess sustained engagement and rehabilitation outcomes over time.

The visual feedback, consistent with previous evidence [53-56], was perceived as intuitive and helpful in increasing awareness of muscle activity. The participants also found that being able to see and control their muscle activity increased their engagement and motivation. Future iterations, as suggested by the end users, will include more engaging interactive games and/or virtual environments, to facilitate long-term use [57-60]. Additionally, more advanced signal processing techniques, such as myoelectric pattern recognition, could be valuable for detecting specific movement patterns and enabling more intuitive control [14,61]. Ease of use and engaging designs have been identified as key factors in increasing treatment adherence [50,62,63] and will therefore be prioritized in the continued development of the training system for self-administered rehabilitation.

The participants also emphasized the need for structured support from the therapist, including regular follow-ups and adjustments to the training program. They highlighted the importance of clear instructions, in written or video formats, to support independent use and long-term adherence. Clear guidance, personalized feedback, and ongoing clinician support are essential for the successful use and sustained engagement with home-based rehabilitation technologies [50,63-65]. Both participants with stroke and clinicians found the training tool suitable for individuals with limited upper limb function but raised some concerns for individuals with limited cognitive capacity or experience with digital tools. On the basis of these insights, future iterations of the training system will focus on incorporating therapist support, clear instructional materials, the potential integration of a virtual coach, and appropriate user targeting to promote effectiveness and broader adoption in home-based rehabilitation.

### Strength and Limitations

The ongoing input from the research partner was a strength of this study, offering valuable feedback that kept the co-design team focused on end users’ needs. The iterative co-design process, supported by the triangulation of interviews, field notes, and questionnaires, as well as ongoing reflexive discussions within the team, strengthened the study’s credibility, transparency, and overall trustworthiness [46,47]. Additionally, interviews, field notes, and a UEQ provided a broader understanding of participants’ experiences.

A strength that also posed a limitation was that some participants were recruited through professional networks and, in many cases, the first author conducted both the sessions and the interviews. While this facilitated access and trust, it may have introduced sampling bias and relationship bias, as prior familiarity could have influenced participants’ responses and the researcher’s interpretation, potentially limiting the diversity and representativeness of perspectives.

The end users’ perceptions were primarily used to inform the design and perceived usefulness of the training system, rather than to evaluate its practical application in clinical settings. The brief, single-session exposure further limited feedback to initial impressions rather than long-term use. Future studies are needed to evaluate the usability, feasibility, and potential effectiveness of the system in people with stroke after a longer training period.

### Conclusions

This study developed and evaluated the usability of a smart textile sEMG biofeedback system designed for self-administered upper limb rehabilitation after stroke. The co-design process, involving a multidisciplinary team of experts and end users, played an important role in identifying users’ needs and preferences, contributing to a more targeted and user-centered design. Individuals with stroke and clinicians found the training tool useful and relevant for rehabilitation. The findings will guide ongoing development and clinical evaluation, but further research is needed to determine the system’s effectiveness as a home-based rehabilitation tool for people with stroke.

## Supplementary material

10.2196/77999 Multimedia Appendix 1 Characteristics of individuals without arm impairment, interview templates, and an example of content analysis from end-user interviews.

10.2196/77999 Multimedia Appendix 2 Characteristics of individuals with stroke and clinicians.

10.2196/77999 Checklist 1 COREQ checklist.

## Abbreviations

COREQ: Consolidated Criteria for Reporting Qualitative Research
sEMG: surface electromyography
UEQ: User Experience Questionnaire
