# Supplementary material for: A Smart Textile Biofeedback Training System for Upper Limb Rehabilitation After Stroke: Co-Design Development and Evaluation Study
Source: JMIR Rehabil Assist Technol. 2026 Apr 13;13:e77999. doi: 10.2196/77999 (PMC13075539; doi:10.2196/77999)
Supplement: Multimedia Appendix 1 [file rehab-v13-e77999-s001.docx]

**Supplementary Material 1**

Characteristics individuals with no arm impairment

|  | Age (years) | Sex | Height (cm) | Weight (kg) | Length forearm (cm) | C. above elbow (cm) | C. below elbow (cm) | C. wrist (cm) |
| --- | --- | --- | --- | --- | --- | --- | --- | --- |
| P1 | 25 | Male | 184 | 77 | 28 | 27 | 27 | 17 |
| P2 | 49 | Male | 187 | 92 | 29 | 29 | 29 | 18 |
| P3 | 30 | Female | 164 | 78 | 24 | 33 | 28 | 17 |
| P4 | 24 | Female | 158 | 66 | 24 | 28 | 24 | 14 |
| P5 | 53 | Female | 171 | 65 | 25 | 24 | 24 | 14 |
| P6 | 31 | Male | 169 | 75 | 24 | 29 | 28 | 17 |
| P7 | 38 | Female | 170 | 67 | 25 | 27 | 24 | 15 |
| P8 | 29 | Female | 166 | 57 | 26 | 24 | 24 | 16 |
| P9 | 44 | Male | 187 | 150 | 29 | 36 | 33 | 19 |
| P10 | 30 | Female | 168 | 79 | 25 | 34 | 30 | 18 |
| P11 | 29 | Female | 166 | 57 | 26 | 24 | 24 | 16 |

C, circumference

**Supplementary Material 2 – Interview demonstration sessions**

**Demo session – Clinicians**

1.Ethics:

Informed consent, photo and video agreement

2. Background information and arm measurements

| Day: | | ID participant: | |
| --- | --- | --- | --- |
| Age: | Sex: | Profession: | |
| Height: | Weight: | Dominant arm: | |
| Length arm: | Upper elbow circumference: | Lower elbow circumference: | Wrist circumference: |
| Size ID: | | | |
| Comments: | | | |

We will start audio recording

| Years of work experience: | Years working with stroke: |
| --- | --- |
| Experience working with sEMG, biofeedback and/or VR?  How familiar are you with tablets and phones? Are you a gamer? Have you use it in rehab? | |
| Comments | |

3. Instructing and testing the textile sleeve

- A researcher will, on itself, show and instruct how to place the textile sleeve and the Shimmer and cables to the sleeve.
- Written introductions on how to place the textile sleeve will be shown to the participant
- Participants will be asked to place the textile sleeve by themselves (therapist will confirm location)
- Participants will also be instructed to share any thoughts they have about the sleeve during the demo and testing process (focus in stroke rehab)

4. Instructing and testing the sEMG-BF software program:

- A researcher will show on itself and instruct how to use the software while the participants will conduct different movements

The tasks would be the following:

- Standard mode: test flex/ext wrist, pronation/supination, open/close hand, thumb and index flex/ext (2 to 3 repetitions per movement)

- Calibration: will be the maximal contraction, and will be a 3 seconds calibration

- Rehabilitation mode: All movements with 5 successful repetitions

- Participants will be asked to operate the software by themselves and describe the task they are doing

The tasks would be the following:

- Standard mode: do 5 repetitions of 2 pair of chosen movements

- Rehabilitation mode: choose 2 pair of movements and conduct calibration and 10 successful repetitions reaching green bar

- Participants will also be instructed to share any thoughts they have about the software during the demo and testing process

**Question end session**

Thinking in stroke rehabilitation:

- How did you experience the feedback showing muscle activity in curves/waves? Was it helpful to you?
- How did you experience the feedback provided through the red and green bar on the side? Was it helpful to you? Any other thoughts?
- Do you think you could use the band longer than the time of the demo (more than 30 min)?
- Adding water on the fabric
- Finding the electrode in the elbow?
- Was the placement of the tablet convenient to visualize the signals?

5. Remove and place sleeve again & thinking out loud:

- Participants will be asked to place and remove the textile sleeve by themselves once more, this time thinking out loud about the task they are performing.

6. Semi-structured interview – Clinicians

Introduction: explain the aim and that there are no right or wrong answers, and that we are interested of any opinion, small or large; inform about the recoding (audio/video)

(Instruction to the interviewer: Start the recoding with ID, date and time)

**Introduction:**

The following interview will focus on your opinion as a therapist/researcher working in stroke rehabilitation. We will ask you to answer the questions while considering how people with upper limb impairment due to stroke may/could/would experience the intervention and training presented in the demo session.

**Overall Impression:**

1. Can you describe your overall thoughts regarding today’s demonstration?

**Textile sleeve:**

1. Can you describe your general experience regarding textile sleeve?
2. What are your thoughts of the possible use of the sleeve in stroke?
3. What was your experience of putting it on and taking it off the sleeve? How do you think will be the case for stroke? Anything that you find particularly useful or challenging?
4. How did you experience wearing the sleeve during the training? How do you think would be the case for a person with a stroke?
5. If you could improve something about the sleeve for stroke rehabilitation, what would it be?
6. Do you think a stroke participant would be able to place and remove the sleeve and cables of the device by themselves at home? What do you think could be challenging?

**Training software and feedback:**

1. Can you describe your general experience regarding the tablet’s interface and software training?
2. What are your thoughts of the possible use of the training software in stroke?
3. Considering the training software/ tablet, what is your impression of the feedback provided on the tablet screen? Anything that you find particularly useful, entertaining or challenging for stroke rehabilitation?
4. If you could improve something about the software/ tablet for stroke rehab what would it be?
5. Do you think a stroke participant would be able to operate the training software/tablet by themselves at home? What do you think could be challenging?

**Future rehabilitation:**

1. Did you felt engage with the demonstration session? Would you use such training method for training stroke participants? Justify your answer
2. Do you think the shown technologies could potentially be a useful tool for upper limb rehabilitation in stroke, and how? Please provide the reason for your answer
3. What improvements or changes would you suggest for using the intervention in self-administered home upper limb stroke rehabilitation?
4. If you could have the access to this intervention in your daily practice , would you use it for stroke rehabilitation? What challenges do you think you could encounter?
5. Do you have any thoughts or opinions about the length of the training session? What would be the best training session length for stroke patients? How often would you suggest the patients to trained?
6. If you could suggest training for home, how often do you think the patients would need support from a therapist? And in what way do you think the support should be provided (physical visit, phone call or video call)?

**Overall closing:**

1. What do you think about the instructions I gave you regarding the sleeve and training software? Were the instructions enough? Was there something that was unclear, or too complicated? Any suggestion for providing same information but for a person with a stroke?
2. Is there anything else you'd like to share?

Thank you for your time and valuable insights today!

7. User-experience questionnaire

**Demo session – Individuals with stroke**

1.Ethics:

Informed consent, photo and video agreement

2. Background information and arm measurements

| Date | | ID participant: | |
| --- | --- | --- | --- |
| Age | Sex: | Profession: | |
| Height: | Weight: | Dominant arm: | |
| Length arm: | Upper elbow circumference: | Lower elbow circumference: | Wrist circumference: |
| Size ID:  Software version:  Comments: | | | |

| Time since stroke: | Type stroke: infarkt or blödning |
| --- | --- |
| Affected side: L or R | |
| Function UL (daily activities):  Sensation UL:  Previous and current UL rehabilitation (any goals)? Training at home (dose)?  Previous experience with sEMG and/or biofeedback?  How comfortable do you feel using tablets and phones? Are you interested in computer/phone games?  Generally, how many things/activities do you usually do in a day? | |
| Comments: | |

3. Instructing and testing the textile sleeve

- A researcher will, on itself, show and instruct how to place the textile sleeve and the Shimmer and cables to the sleeve.
- Written introductions on how to place the textile sleeve will be shown to the participant
- Participants will be asked to place the textile sleeve by themselves (therapist will confirm location)
- Participants will also be instructed to share any thoughts they have about the sleeve during the demo and testing process (focus in stroke rehab)

4. Instructing and testing the sEMG-BF software program:

- A researcher will show on itself and instruct how to use the software while the participants will conduct different movements

The tasks would be the following:

- Standard mode: test flex/ext wrist, pronation/supination, open/close hand, thumb and index flex/ext (2 to 3 repetitions per movement)

- Calibration: will be the maximal contraction, and will be a 3 seconds calibration

- Rehabilitation mode: All movements with 5 successful repetitions

- Participants will be asked to operate the software by themselves and describe the task they are doing

The tasks would be the following:

- Standard mode: do 5 repetitions of 2 pair of chosen movements

- Rehabilitation mode: choose 2 pair of movements and conduct calibration and 10 successful repetitions reaching green bar

- Participants will also be instructed to share any thoughts they have about the software during the demo and testing process

5. Remove and place sleeve again & thinking out loud:

- Participants will be asked to place and remove the textile sleeve by themselves once more, this time thinking out loud about the task they are performing.

6. Semi-structured interview – Individuals with stroke

Introduction: explain the aim and that there are no right or wrong answers, and that we are interested of any opinion, small or large; inform about the recoding (audio/video)

(Instruction to the interviewer: Start the recoding with ID, date and time)

**Overall:**

1. What is your general impression about this form of exercise?

**Sleeve:**

1. Now, I will ask you some questions about the sleeve. What are your overall thoughts on it?
2. How was your experience putting the sleeve on and taking it off by yourself? And how was it to wear it during the session?

**Tablet:**

1. Now, I will ask you some questions about what we did on the tablet/screen. What are your overall thoughts on it?
2. How was it for you to see your own muscle activity on the screen? And how was your experience controlling the color bars?
3. In the beginning we calibrated the system in different ways (name the ways we try), what are your thoughts on this step? Would this be something that you could manage to do by yourself?
4. How did you experience the elements showed on the screen? Do you have any preferences for sizes or colors for these elements? Now you have used a tablet, did this work for you? Would you prefer some other way?

**Home:**

1. If you had this system in your home, is it something you would use after you have learned how to use it? Do you see anything that might be problematic? Do you think you would need support or help from a therapist or someone else?
2. Do you think you could have used this system earlier during your rehabilitation if you had access to it?

**Overall closure:**

1. Overall, what do you think about this way of training?
2. Overall, based on today's session, do you think the instructions I provided were clear?
3. Now to finish, is there anything else that you would like to add?

7.Assessments:

- User-experience questionnaire in Swedish
- FMA-UE total score: _______
- FMA-UE sensory: ____
- MAS:_______

**Supplementary Material 3 – Example of content analysis from end-user interviews**

| **Meaning units** | **Condensed meaning unit** | **Codes** | **Subcategories** | **Categories** |
| --- | --- | --- | --- | --- |
| *S6: Uh, I find it [the training system] interesting... with I think much potential. And it's because you have to take some control over your muscles, you feel that you have no control, or very little control. I think that can help you to understand your muscle and have some more control.* | The tool seems exciting and motivating because it helps individuals with stroke reconnect with and control their muscles. | Motivated by the biofeedback. | Rewarding and motivating to see my muscle activity. | This could be a new exciting training tool for stroke rehabilitation. |
| *C1: I don't think the sleeve is the problem [for self-administrated training]. I think the technical use is more complicated with the app... and not the sleeve.* | The software is more difficult to use independently than the sleeve. | The independent use of the software is a concern. | More is needed for independent long-term use | The training tool works fine – some changes would enhance independent training. |

S: Individual with stroke; C: Clinician.
