# Supplementary material for: A Smart Textile Biofeedback Training System for Upper Limb Rehabilitation After Stroke: Co-Design Development and Evaluation Study
Source: JMIR Rehabil Assist Technol. 2026 Apr 13;13:e77999. doi: 10.2196/77999 (PMC13075539; doi:10.2196/77999)
Supplement: Multimedia Appendix 2 [file rehab-v13-e77999-s002.docx]

| *Table S1: Characteristics of individuals with stroke* | | | | | | | | | | |
| --- | --- | --- | --- | --- | --- | --- | --- | --- | --- | --- |
|  | **S1** | **S2** | **S3** | **S4** | **S5** | **S6** | **S7** | **S8** | **S9** | **S10** |
| Age (years) | 61 | 19 | 80 | 73 | 53 | 68 | 80 | 55 | 74 | 62 |
| Sex | Female | Male | Female | Female | Male | Female | Female | Female | Male | Male |
| Time since stroke (years) | 28 | 0.5 | 1 | 1.58 | 3.58 | 16 | 0.42 | 3.25 | 5.25 | 6.5 |
| Type of stroke | Hemorrhage | Hemorrhage | Ischemic | Hemorrhage | Hemorrhage | Ischemic | Ischemic | Hemorrhage | Hemorrhage | Ischemic |
| Dominant side | Right | Right | Right | Right | Right | Ambidextrous | Right | Right | Right | Right |
| Affected side | Left | Left | Right | Right | Left | Left | Right | Left | Left | Right |
| FMA-UE motor function  (max score 66) | 9 | 15 | 55 | 41 | 51 | 17 | 19 | 14 | 18 | 58 |
| FMA-UE sensation  (max score 12) | 4 | 4 | 12 | 2 | 12 | 12 | 12 | 7 | 3 | 12 |
| MAS (sum) | 11 | 11 | 0 | 2 | 3 | 7 | 5 | 7 | 9 | 2 |
| Size sleeve | Small | Medium | Medium | Large | Large | Large | Medium | Large | Large | Large |

FMA-UE, Fugl-Meyer Assessment of Upper Extremity; MAS, Modify the Ashworth Scale

| *Table S2: Clinicians characteristics* | | | | | | | |  |  |
| --- | --- | --- | --- | --- | --- | --- | --- | --- | --- |
|  | **C1** | **C2** | **C3** | **C4** | **C5** | **C6** | **C7** | **C8** | **C9** |
| Age (years) | 58 | 37 | 24 | 24 | 31 | 35 | 61 | 39 | 60 |
| Sex | Female | Female | Female | Female | Female | Female | Male | Male | Female |
| Profession | PT | PT | PT | OT | OT | Rehab doctor | PT | PT | PT |
| Years of experience in stroke rehab | 25 | 6.5 | 1 | 1 | 1 | 6 | 20 | 6 | 30 |
| Work settings | Inpatient | Inpatient | Inpatient | Inpatient | In-outpatient | In-outpatient | In-outpatient | In-outpatient | Inpatient |
| Size sleeve | Large | Large | Large | Small | Large | Large | Large | Large | Large |

PT, Physiotherapist; OT, occupational therapist
